# Environmental stresses suppress nonsense-mediated mRNA decay (NMD) and affect cells by stabilizing NMD-targeted gene expression

**DOI:** 10.1038/s41598-018-38015-2

**Published:** 2019-02-04

**Authors:** Fusako Usuki, Akio Yamashita, Masatake Fujimura

**Affiliations:** 1 Department of Clinical Medicine, National Institute for Minamata Disease, 4058-18 Hama, Minamata, 867-0008 Japan; 2 Department of Molecular Biology, Yokohama City University School of Medicine, 3-9 Fuku-ura, Kanazawa, Yokohama 236-0004 Japan; 3 Basic Medical Sciences, National Institute for Minamata Disease, 4058-18 Hama, Minamata, 867-0008 Japan

## Abstract

Nonsense-mediated mRNA decay (NMD) is a cellular mechanism that eliminates mRNAs that harbor premature translation termination codons (PTCs). Here, we investigated the effects of environmental stresses (oxidative stress and endoplasmic reticulum (ER) stress) on NMD activity. Methylmercury (MeHg) was used to cause oxidative stress and thapsigargin to stress the ER. NMD suppression, evidenced by upregulation of NMD-sensitive mRNAs and a decrease in UPF1 phosphorylation, was observed in MeHg-treated myogenic cells, cerebral cortical neuronal cells, and astroglial cells. Mild ER stress amplified NMD suppression caused by MeHg. To elucidate the cause of stress-induced NMD suppression, the role of the phospho-eIF2α/ATF4 pathway was investigated. Knockdown and non-phosphorylatable eIF2α-transfection studies demonstrated the critical role of phospho-eIF2α-mediated repression of translation in mild ER stress-induced NMD suppression. However, NMD suppression was also observed in phospho-eIF2α-deficient cells under mild ER stress. Mechanistic target of rapamycin suppression-induced inhibition of cap-dependent translation, and downregulation of the NMD components UPF1, SMG7, and eIF4A3, were probably involved in stress-induced NMD suppression. Our results indicate that stress-induced NMD suppression has the potential to affect the condition of cells and phenotypes of PTC-related diseases under environmental stresses by stabilizing NMD-targeted gene expression.

## Introduction

Nonsense codons that prematurely interrupt an in-frame sequence termed the premature translation termination codons (PTCs) are normally eliminated by nonsense-mediated mRNA decay (NMD)^1–4^. Targets for NMD can include mutationally- or transcriptionally-induced nonsense or frameshift codons, upstream open reading frames (uORFs), alternatively spliced or mis-spliced mRNA, and the UGA codon for selenocystein under selenium deficiency^5^. Traditionally, NMD has been considered an mRNA quality surveillance mechanism to protect an organism against deleterious dominant-negative or gain-of-function effects of truncated proteins that arise from PTCs. However, some truncated proteins retain normal functions, at least partially^6–8^. If NMD down-regulates aberrant proteins that retain some normal cell function, detrimental effects of mutation can be exacerbated^9–13^.

It has also been demonstrated that several stress-induced genes possessing uORFs, or other features prone to PTCs, such as alternatively spliced transcripts, are targeted by NMD, the inhibition of which stabilizes their cognate mRNAs and augments the cellular stress responses^14–16^. We previously showed that endoplasmic reticulum (ER) stress preconditioning protects cells against cytotoxicity of methylmercury (MeHg), a major environmental toxicant^17^. The underlying mechanism is the induction of integrated stress responses, including NMD suppression, phosphorylation of eukaryotic initiation factor 2 alpha (eIF2α), accumulation of activating transcription factor 4 (ATF4), and upregulation of stress-related proteins. We hypothesized that environmental stresses suppressing NMD might thereby affect the expression of truncated proteins that arise from PTCs.

Here, we aimed to investigate the effects of two environmental stresses, oxidative stress and mild ER stress, on NMD activity in the mouse MeHg-susceptible myogenic cell line C2C12-DMPK160^18,19^ and rat central nervous system cells [cerebral cortical neuronal cells (CNCs) and astroglial cells (AGCs)]. NMD and the change in NMD that occurs upon exposure to stresses in the central nervous system are not clear yet. Our results demonstrated that environmental stresses induce NMD suppression in aforementioned cells, suggesting that this may be a mechanism through which these stresses affect cellular condition. We further investigated the mechanism of NMD suppression induced by mild ER stress using mutant cells expressing non-phosphorylatable eIF2α. We demonstrated that phospho-eIF2α-mediated repression of translation plays a critical role, and that mechanistic target of rapamycin (mTOR) suppression-induced inhibition of cap-dependent translation, and downregulation of the NMD components UPF1, SMG7, and eIF4A3 are also involved in environmental stress-induced NMD suppression.

## Results

### Environmental stresses suppress NMD in a variety of cells

**Figure 1:**
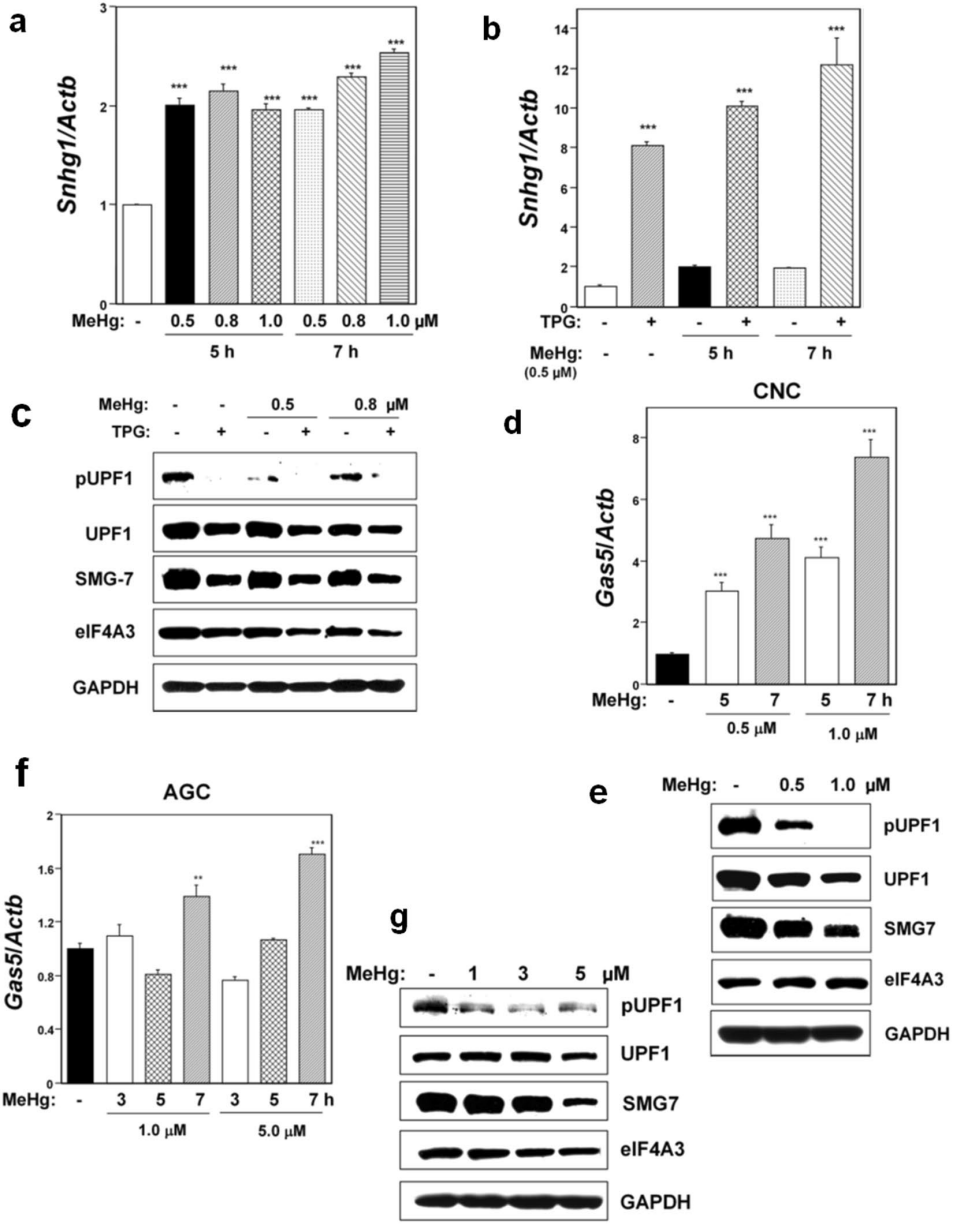
Effects of MeHg or mild ER stress on NMD activity in C2C12-DMPK160 cells (**a**–**c**), CNCs (**d**,**e**), and AGCs (**f**,**g**). (**a**) RT-qPCR analysis of *Snhg1* mRNA. The histogram depicts *Snhg1* mRNA normalized to *Actb* mRNA presented as the fold-increase over non-pretreated controls. Values represent mean ± SE of three separate experiments. ***Significantly different from MeHg-untreated cells by one-way ANOVA followed by Bonferroni’s multiple comparison test (p < 0.001). (**b**) Effects of mild ER stress on NMD activity. C2C12-DMPK160 cells pretreated with TPG (0.2 μg/ml) for 16 h were exposed to 0.5 μM MeHg for 5 or 7 h. The histogram depicts *Snhg1* mRNA normalized to *Actb* mRNA presented as the fold-increase over non-pretreated MeHg-untreated controls. Values represent mean ± SE (n = 3). ***Significantly different from TPG-untreated cells by one-way ANOVA followed by Bonferroni’s multiple comparison test (p < 0.001). (**c**) Western blotting analyses of NMD components’ protein expression. Total cell lysates prepared 7 h after exposure to 0.5 or 0.8 μM MeHg were analyzed using the indicated antibody probes. Cropped blots are shown; all gels were run under the same experimental conditions. Raw data are shown in Supplementary Fig. 1c with dividing lines and with quantitative data. (**d**,**f**) RT-qPCR analysis of *Gas5* mRNA. The histogram depicts *Gas5* mRNA normalized to *Actb* mRNA represented as the fold-increase over MeHg-untreated controls. Values shown are the mean ± SE of three separate experiments. **^,^***Significantly different from MeHg-untreated cells by one-way ANOVA followed by Bonferroni’s multiple comparison test (**p < 0.01, ***p < 0.001). (**e**,**g**) Western blotting analyses of expression of NMD component proteins. Total cell lysates prepared 24 h for CNCs or 9 h for AGCs after exposure to the indicated concentration of MeHg were analyzed using the indicated antibody probes. Cropped blots are shown; all gels were run under the same experimental conditions. Raw data are shown in Supplementary Fig. 1e, g with dividing lines and with quantitative data.

We investigated the effects on NMD activity of oxidative stress and mild ER stress in mouse MeHg-susceptible myogenic C2C12-DMPK160 cells, rat CNCs, and rat AGCs. MeHg (0.5–1.0 μM) was used as an oxidative stressor^5,18,20^ and the ER Ca^2+^-ATPase inhibitor, thapsigargin (TPG, supplied at 0.2 μg/ml) was used as a mild ER stressor^17^. The critical role of oxidative stress in the pathogenesis of MeHg cytotoxicity has been clarified both *in vitro*^5,18–23^ and *in vivo*^10,24,25^. We have also reported that MeHg-intoxication can be triggered by the occurrence of oxidative stress at the early phase using C2C12-DMPK160 cells^20^. Increased levels of reactive oxygen species have been demonstrated in rat CNCs and AGCs after exposure to MeHg^26–28^. Suppression of NMD was evidenced by upregulation of NMD target non-protein-coding small nucleolar RNA host gene 1 (*Snhg1*) or growth arrest-specific 5 (*Gas5*) mRNA harboring PTCs. We have demonstrated the effects of depleting NMD effectors (SMG1 and SMG7) on the upregulation of *Snhg1* mRNAs during ER stress^29^. As a further confirmation of NMD suppression, we evaluated UPF1 phosphorylation (p-UPF1) since the UPF1 phosphorylation-dephosphorylation cycle is essential for NMD^30,31^. As shown in Fig. 1a and b, treatment with MeHg upregulated *Snhg1* mRNA, and pretreatment with TPG 16 h before MeHg exposure (ER stress preconditioning) further amplified this upregulation of *Snhg1* mRNA in C2C12-DMPK160 cells. Western blot analyses confirmed the downregulation of p-UPF1 in MeHg-treated cells and its amplification in TPG-pretreated and MeHg-treated cells compared to control cells (Fig. 1c).

NMD suppression in rat CNCs and AGCs was investigated by assessing the expression of *Gas* mRNA, since their *Snhg1* mRNA expression was weak. Notably, the effect of MeHg treatment on *Gas5* mRNA expression was different between CNCs and AGCs. NMD suppression was clearly observed in MeHg-treated CNCs, as evidenced by upregulation of *Gas5* mRNA and downregulation of p-UPF1 compared to control cells (Fig. 1d,e). However, NMD suppression was weak in MeHg-treated AGCs (Fig. 1f,g). In addition, western blot analyses demonstrated a decrease in the expression of the NMD components UPF1 and SMG7 in all cells examined, and eIF4A3 in C2C12-DMPK160 cells (Fig. 1c,e,g). These results indicated that NMD suppression varied in the different cell types and conditions.

### Causative factors of NMD suppression induced by mild ER stress

**Figure 2:**
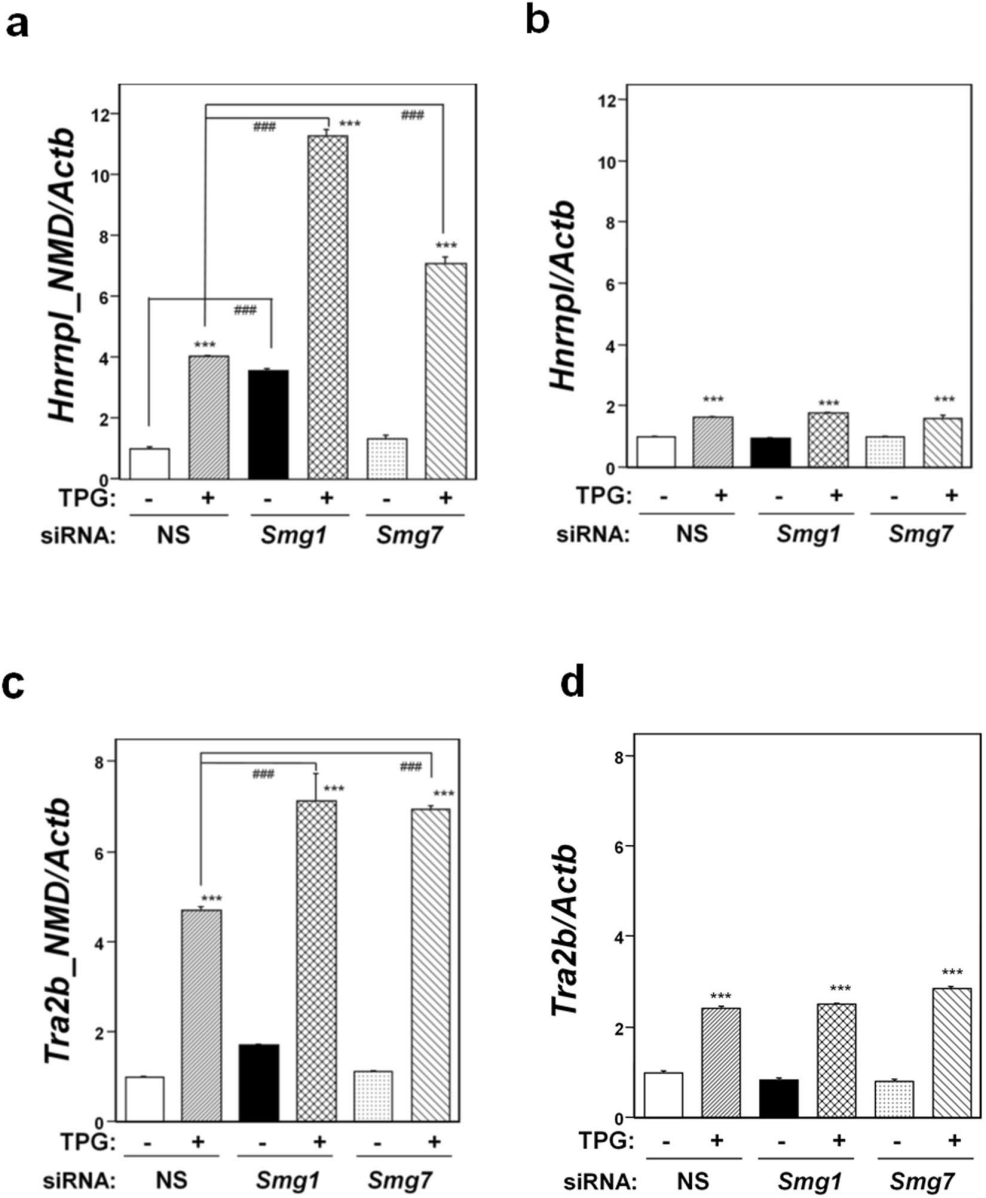
Effects of depleting NMD effectors on NMD-sensitive and NMD-non-sensitive isoform mRNA expression under mild ER stress. NS, non-silencing. (**a**,**c**) RT-qPCR analysis of NMD-sensitive mRNA of *Hnrnpl_NMD* (**a**) and *Tra2b_NMD* (**c**). Total RNA was extracted from untreated cells or following treatment with 0.2 μg/ml TPG for 16 h. The histogram depicts *Hnrnpl_NMD or Tra2b_NMD* mRNA normalized to *Actb* mRNA presented as the fold-increase over non-pretreated NS siRNA-transfectants. Values shown are the mean ± SE of three separate experiments. ***Significantly different from TPG-untreated cells by one-way ANOVA followed by Bonferroni’s multiple comparison test (p < 0.001). ^###^Significantly different from NS siRNA-transfectants by one-way ANOVA followed by Bonferroni’s multiple comparison test (p < 0.001). (**b**,**d**) RT-qPCR analysis of mRNA of NMD-non-sensitive *Hnrnpl* (**b**) or *Tra2b* (**d**) isoform using the same data set of NMD-sensitive isoform mRNA of panel a or c, to determine expression under mild ER stress. The histogram depicts each mRNA normalized to *Actb* mRNA presented as the fold-increase over non-pretreated NS siRNA-transfectants. Values shown are the mean ± SE of three separate experiments. ***Significantly different from TPG-untreated cells by one-way ANOVA followed by Bonferroni’s multiple comparison test (p < 0.001).

Next, we investigated the causal factors involved in NMD suppression in C2C12-DMPK160 cells under mild ER stress. Since the phospho-eIF2α/ATF4 pathway is a key transcriptional activator involved in the adaptation to ER stresses, we first investigated the role of ATF4 or phospho-eIF2α in environmental stress-induced NMD suppression. NMD suppression was investigated by RT-qPCR analysis of the NMD-sensitive isoform of heterogenous nuclear ribonucleoprotein L (Hnrnpl_NMD)^32^ and western blot analysis for phosphorylation of UPF1. To confirm that the upregulation of NMD-sensitive *Hnrnpl_NMD* mRNA was not caused by transcriptional change, but rather by the true effect of NMD suppression, we firstly investigated the effects of depleting NMD effectors (SMG1 and SMG7) on the expression of NMD-sensitive *Hnrnpl_NMD* and NMD-non-sensitive *Hnrnpl* isoform mRNA expression during stress. As shown in Fig. 2a and b, depleting the NMD effectors resulted in the upregulation of the NMD-sensitive isoform mRNA, but not NMD-non-sensitive isoform mRNA, under ER stress. The same findings were obtained with another gene set (*Tra2b_NMD and TRa2b)* (Fig. 2c,d). The results indicated that the observed NMD-sensitive *Hnrnpl_NMD* isoform mRNA upregulation is not a transcriptional effect, but rather represents true NMD suppression.

**Figure 3:**
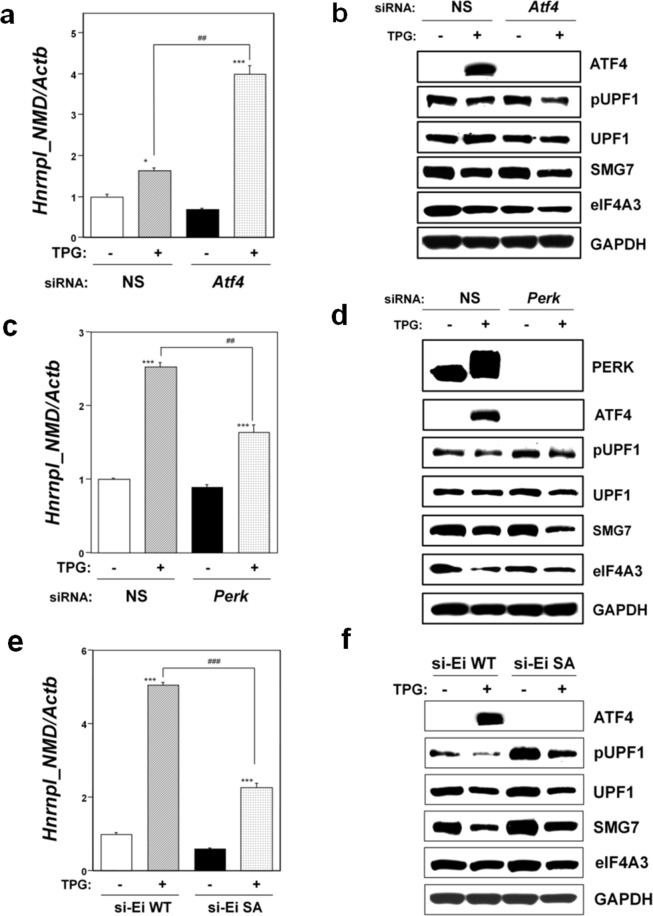
Effects of the phospho-eIF2α/ATF4 pathway on NMD activity under ER stress. NS, non-silencing. (**a**,**c**) Effects of *Atf4* (**a**) or *Perk* (**c**) knockdown on *Hnrnpl_NMD* mRNA expression were analyzed by RT-qPCR. Total RNA was extracted from untreated cells or following treatment with 0.2 μg/ml TPG for 16 h. The histogram depicts *Hnrnpl_NMD* mRNA normalized to *Actb* mRNA presented as the fold-increase over non-pretreated NS siRNA-transfectants. Values represent mean ± SE of three separate experiments. *^,^***Significantly different from TPG-untreated cells by one-way ANOVA followed by Bonferroni’s multiple comparison test (*p < 0.05, ***p < 0.001). ^###^Significantly different from TPG-treated NS siRNA-transfectants by one-way ANOVA followed by Bonferroni’s multiple comparison test (p < 0.001). (**b**,**d**) Effects of *Atf4* (**b**) or *Perk* (**d**) knockdown on NMD components expression. Cell lysates were prepared 16 h after exposure to 0.2 μg/ml TPG. Cropped blots are shown; all gels were run under the same experimental conditions. Raw data are shown in Supplementary Fig. 3b and d with dividing lines and with quantitative data. (**e**) Effects of eIF2α phosphorylation on *Hnrnpl_NMD* mRNA expression were analyzed by RT-qPCR. WT, cell line transfected with wild-type eIF2α plasmid. SA, cell line transfected with mutant eIF2α-SA plasmid. Endogenous eIF2α was knocked down by transfection with synthetic siRNA against eIF2α (si-Ei). Total RNA was extracted from untreated cells or following treatment with 0.2 μg/ml TPG for 16 h. The histogram depicts *Hnrnpl_NMD* mRNA normalized to *Actb* mRNA presented as the fold-increase over non-pretreated WT control. Values represent mean ± SE of three separate experiments. ***Significantly different from TPG-untreated cells by one-way ANOVA followed by Bonferroni’s multiple comparison test (p < 0.001). ^###^Significantly different from TPG-treated WT cell line by one-way ANOVA followed by Bonferroni’s multiple comparison test (p < 0.001). (**f**) Effect of eIF2α phosphorylation on NMD components expression under mild ER stress. Cells are as shown in (**e**). Cell lysates were prepared 16 h after exposure to 0.2 μg/ml TPG. Cropped blots are shown; all gels were run under the same experimental conditions. Raw data are shown in Supplementary Fig. 3f with dividing lines and with quantitative data.

To investigate the role of ATF4 or phospho-eIF2α in environmental stress-induced NMD suppression, we used small interfering RNA (siRNA) technology to knockdown ATF4 or the eIF2α phosphorylation kinase protein kinase R-like ER kinase (PERK). Both are activated as a main kinase during ER stress. As shown in Fig. 3a, mild ER stress upregulated *Hnrnpl_NMD* mRNA in both non-silencing (NS) siRNA-transfectants and *Atf4* knockdown cells, suggesting that NMD was suppressed in these TPG-treated cells. However, the level of *Hnrnpl_NMD* mRNA was significantly upregulated in *Atf4* knockdown cells compared to NS siRNA-transfectants. Western blot analysis demonstrated downregulation of phospho-UPF1, SMG7, and eIF4A3 in *Atf4* knockdown cells compared to NS siRNA-transfectants under mild ER stress (Fig. 3b). In contrast, *Perk* knockdown cells treated with TPG showed significantly downregulated *Hnrnpl_NMD* mRNA expression compared to TPG-treated, non-silencing siRNA-transfectants (Fig. 3c). Western blot analysis did not demonstrate downregulation of phospho-UPF1 in *Perk* knockdown cells compared to NS siRNA-transfectants under mild ER stress (Fig. 3d). These findings indicated that PERK expression is an upstream effector of NMD suppression, but ATF4 is not. The collective findings indicated that phosphorylation of eIF2α plays a role in the induction of NMD suppression under mild ER stress.

In order to further determine the role of phosphorylation of eIF2α in NMD suppression under mild ER stress, we used previously established stable wild-type phosphorylatable (WT) and mutant non-phosphorylatable (SA) eIF2α-transfected cell lines^29^. Internal eIF2α in both established cell lines was knocked down by the transfection of siRNA targeted to internal eIF2α, as previously reported^29^. Significant downregulation of ATF4 was observed in SA cells compared to WT cells (Fig. 3f). As shown in Fig. 3e, mild ER stress upregulated *Hnrnpl_NMD* mRNA in both WT and SA cells, but the level was significantly downregulated in SA cells compared with WT cells. Western blot analysis demonstrated the reduced downregulation of phospho-UPF1, UPF1, SMG7, and eIF4A3 in SA cells compared to WT cells under mild ER stress (Fig. 3f). These results confirmed that phosphorylation of eIF2α plays a role in NMD suppression under mild ER stress. However, significant upregulation of *Hnrnpl_NMD* mRNA and downregulation of phospho-UPF1 were also observed in the TPG-treated mutant SA cells compared to SA cells that were not treated with TPG. These results suggested that NMD suppression might be also induced by factors other than phospho-eIF2α-mediated translation suppression in SA cells exposed to mild ER stress, although a reduction in the expression of UPF1, SMG7, and eIF4A3 induced by mild ER stress might also contribute to NMD suppression.

### Effect of mTOR expression on NMD suppression in TPG-treated mutant SA cells

**Figure 4:**
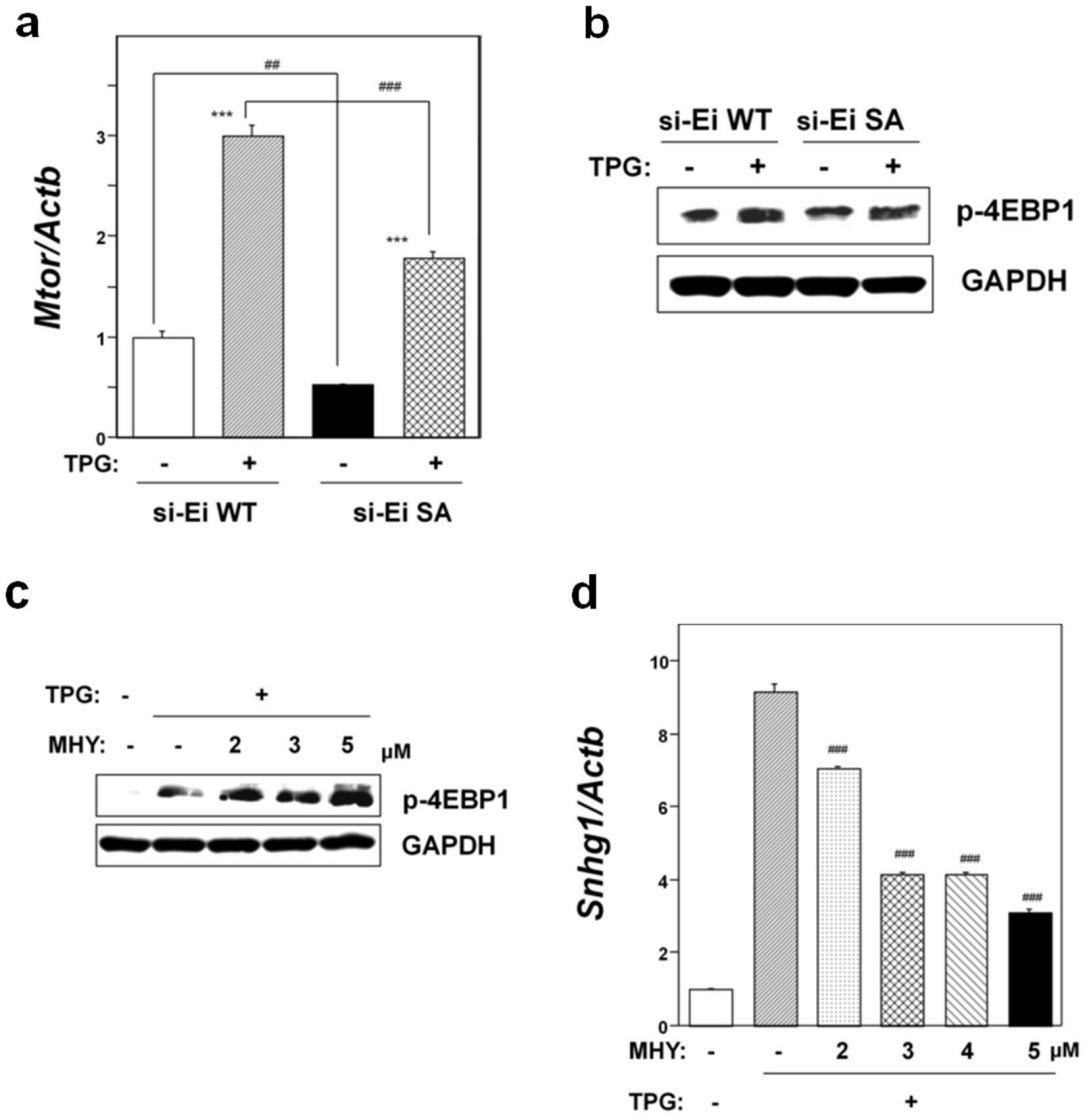
Effects of eIF2α phosphorylation on mTORC1 signaling. WT, cell line transfected with wild-type eIF2α plasmid; SA, cell line transfected with mutant eIF2α-SA plasmid. Endogenous eIF2α was knocked down by transfection with synthetic siRNA against eIF2α (si-Ei). (**a**) RT-qPCR analysis of *Mtor* mRNA. Total RNA was extracted from untreated cells or following treatment with 0.2 μg/ml TPG for 16 h. The histogram depicts *Mtor* mRNA normalized to *Actb* mRNA presented as the fold-increase over non-pretreated WT control. Values represent mean ± SE of three separate experiments. ***Significantly different from TPG-untreated cells by one-way ANOVA followed by Bonferroni’s multiple comparison test (p < 0.001). ^##,###^Significantly different from WT cell line by one-way ANOVA followed by Bonferroni’s multiple comparison test (^##^p < 0.01, ^###^p < 0.001). (**b**) Western blotting analysis for phospho-4EBP1 (p-4EBP1). Total cell lysates were prepared 16 h after exposure to 0.2 μg/ml TPG. Cropped blots are shown; all gels were run under the same experimental conditions. Raw data were shown in Supplementary Fig. 4b with dividing lines and with quantitative data. (**c**) Effect of the mTOR activator MHY1485 (MHY) on expression of p-4EBP1. SA cells were treated with the indicated concentration of MHY for 5 h before cell preparation. Total cell lysates were prepared 16 h after exposure to 0.2 μg/ml TPG. Cropped blots are shown; all gels were run under the same experimental conditions. Raw data are shown in Supplementary Fig. 4c with dividing lines and with quantitative data. (**d**) Effect of mTOR activator MHY1485 (MHY) on NMD activity. Expression of *Snhg1* mRNA was evaluated by RT-qPCR. Total RNA was extracted from SA cells 16 h after exposure to 0.2 μg/ml TPG. SA cells were treated with the indicated concentration of MHY for 3 h before RNA extraction. The histogram depicts *Snhg1* mRNA normalized to *Actb* mRNA presented as the fold-increase over MHY- and TPG-untreated control. Values shown are the mean ± SE of three separate experiments. ^###^Significantly different from TPG-treated MHY-non-treated cells by one-way Welch’s *t*-test (p < 0.001).

To further investigate the causes of NMD suppression in the mutant SA-preconditioned cells, we examined mTOR expression in this cell line. As shown in Fig. 4a, mTOR mRNA expression was significantly suppressed in non-preconditioned SA cells compared to non-preconditioned WT cells. Although pretreatment with TPG upregulated mTOR mRNA expression in both WT and SA cells, the level was significantly lower in SA cells (p < 0.001). Western blot analysis for phospho T37/46 of 4EBP1 (p-4EBP1), a direct substrate of mTORC1, revealed the lower expression of p-4EBP1 in SA cells compared to WT cells under mild ER stress, suggesting the down-regulation of mTORC1 activity (Fig. 4b). These results demonstrated that phosphorylation of eIF2α is an upstream effecter of the mTOR pathway.

Next, we investigated the effect of mTOR pathway activation on NMD activity to determine the role of mTORC1 in NMD suppression in mutant non-phosphorylatable eIF2α-transfectants exposed to mild ER stress. This rescue experiments involving the expression of mTORC1 was done using the mTOR activator, MHY1485^33^. Western blot analysis revealed the dose-dependent upregulation of p-4EBP1 in MHY1485-treated SA cells under mild ER stress (Fig. 4c). Expression analysis by RT-qPCR in SA cells revealed that MHY1485 treatment significantly repressed TPG-preconditioning induced accumulation of *Snhg1* mRNA in a dose-dependent manner (Fig. 4d). The findings suggested that the mTORC1 pathway plays a role in NMD suppression in mutant non-phosphorylatable eIF2α-transfectants under mild ER stress, possibly by the inhibition of cap-dependent mRNA translation.

### Role of the mTOR pathway in integrated stress responses induced by mild ER stress

**Figure 5:**
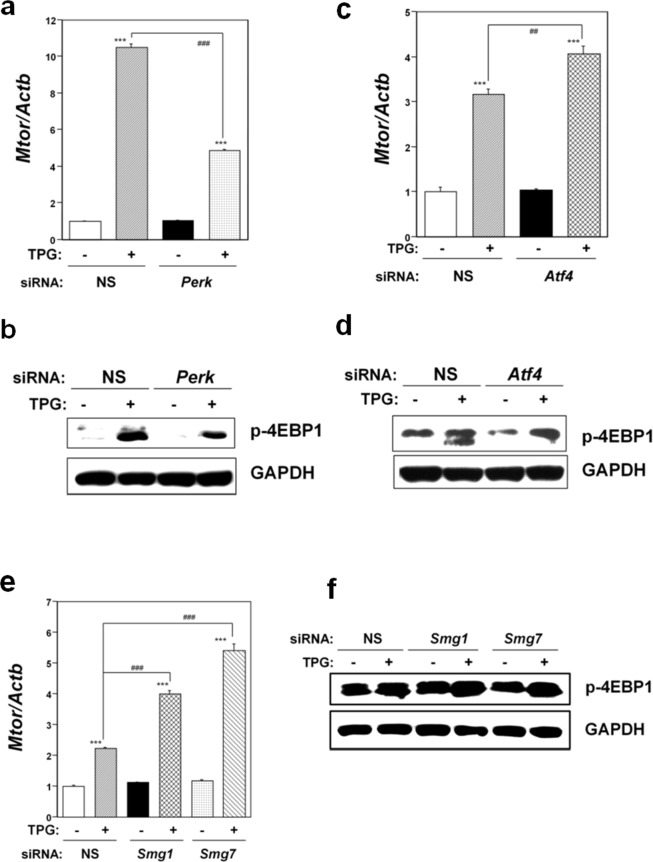
Effect of *Perk* knockdown (**a**,**b**), *Atf4* (**c**,**d**) or the NMD components *Smg-1* or *Smg-7* (**e**,**f**) on mTORC1 signaling. NS, non-silencing. (**a**,**c**,**e**) Analysis of *Mtor* mRNA by RT-qPCR. Total RNA was extracted from untreated cells or following treatment with 0.2 μg/ml TPG for 16 h. The histogram depicts *Mtor* mRNA normalized to *Actb* mRNA presented as the fold-increase over non-pretreated NS siRNA-transfectants. Values shown are the mean ± SE of three separate experiments. ***Significantly different from TPG-untreated cells by one-way ANOVA followed by Bonferroni’s multiple comparison test (p < 0.001). ^##,###^Significantly different TPG-treated NS siRNA-transfectants by one-way ANOVA followed by Bonferroni’s multiple comparison test (^##^p < 0.01, ^###^p < 0.001). (**b**,**d**,**f**) Western blotting analysis for phospho-4EBP1 (p-4EBP1). Total cell lysates were prepared 16 h after exposure to 0.2 μg/ml TPG. The levels of depletion of SMG1 and SMG7 using *Smg1* and *Smg7* siRNAs are shown in Supplementary Fig. 5f. Cropped blots are shown; all gels were run under the same experimental conditions. Raw data were shown in Supplementary Fig. 5b,d,f with dividing lines and with quantitative data.

To investigate the role of the mTOR pathway in integrated stress responses induced by mild ER stress, we performed knockdown experiments. As shown in Fig. 5a, significant *Mtor* mRNA downregulation was observed in TPG-treated, *Perk* knockdown C2C12-DMPK160 cells compared to TPG-treated, NS siRNA-transfectants, indicating that PERK is an upstream effector of the mTOR pathway under mild ER stress. Western blot analysis for p-4EBP1 demonstrated downregulation of the mTORC1 pathway under these conditions (Fig. 5b). In contrast, significant *Mtor* mRNA upregulation was observed in TPG-treated, *Atf4*-knockdown or NMD-suppressed cells compared to TPG-treated, non-silencing siRNA-transfectants (Fig. 5c,e). The findings indicated that the expression of mTOR is not a downstream effector of ATF4 expression or NMD suppression under mild ER stress. Western blot analysis for p4EBP1 demonstrated activation of the mTOR pathway under these conditions (Fig. 5d,f).

### Effect of *Mtor* knockdown on NMD

**Figure 6:**
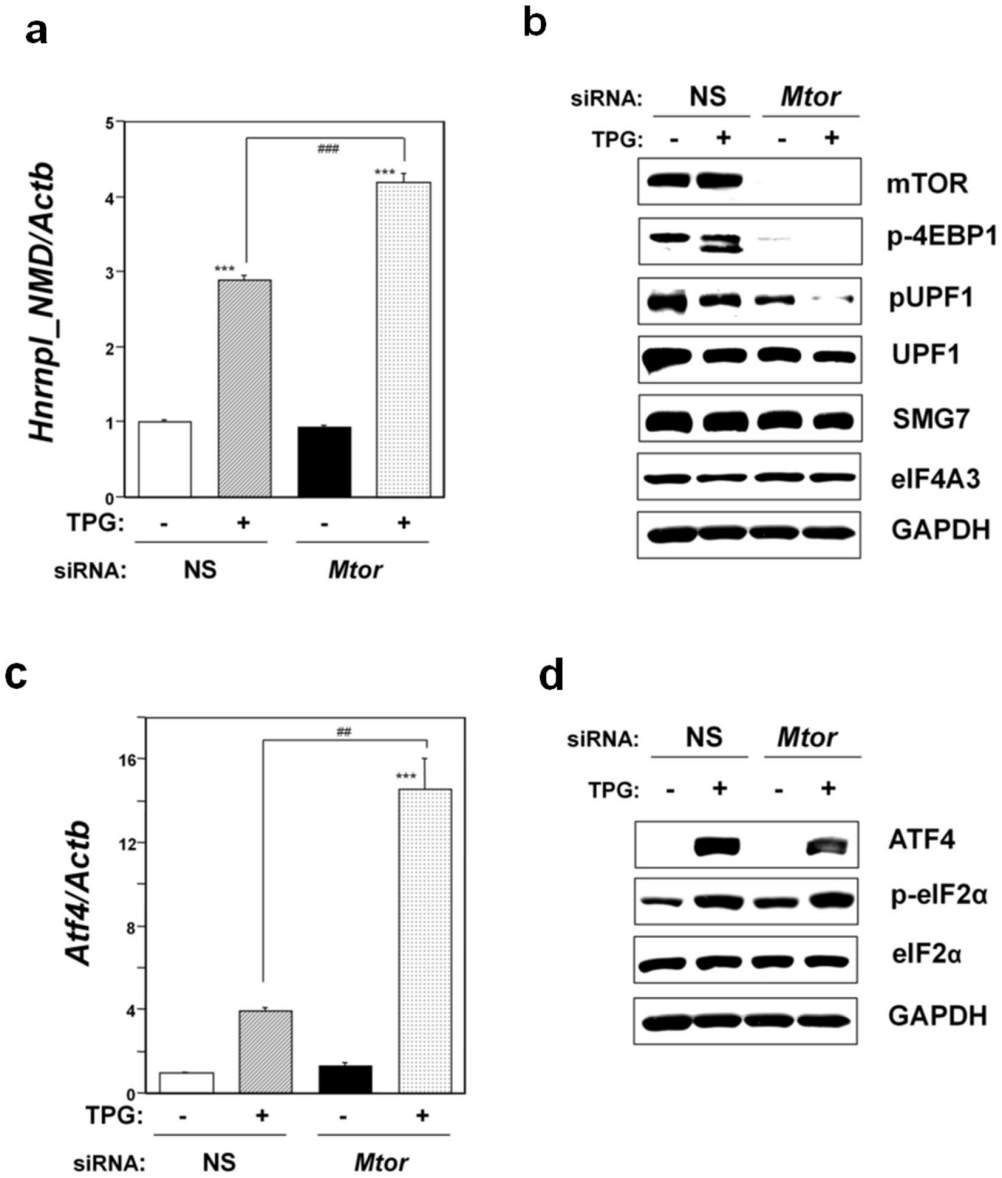
Effects of *Mtor* knockdown on NMD activity (**a**,**b**), ATF4 expression (**c**,**d**). (**a**) Analysis of *Hnrnpl_NMD* mRNA by RT-qPCR. Total RNA was extracted from cells treated with 0.2 μg/ml TPG for 16 h. The histogram depicts *Hnrnpl_NMD* mRNA normalized to *Actb* mRNA presented as the fold-increase over non-pretreated NS siRNA-transfectants. Values shown are the mean ± SE of three separate experiments. ***Significantly different from TPG-untreated cells by one-way ANOVA followed by Bonferroni’s multiple comparison test (p < 0.001). ^###^Significantly different from TPG-treated NS siRNA-transfectants by one-way ANOVA followed by Bonferroni’s multiple comparison test (p < 0.001). (**b**) Western blotting analyses of expression of mTOR, p-4EBP1, and NMD components. Total cell lysates prepared 16 h after exposure to 0.2 μg/ml TPG were analyzed using the indicated antibody probes. Cropped blots are shown; all gels were run under the same experimental conditions. Raw data are shown in Supplementary Fig. 6b with dividing lines and with quantitative data. (**c**) Analysis of *Atf4* mRNA by RT-qPCR. Total RNA was extracted from cells treated with 0.2 μg/ml TPG for 16 h. The histogram depicts *Atf4* mRNA normalized to *Actb* mRNA presented as the fold-increase over non-pretreated NS siRNA-transfectants. Values shown are the mean ± SE of three separate experiments. ***Significantly different from TPG-untreated cells by one-way ANOVA followed by Bonferroni’s multiple comparison test (p < 0.001). ^##^Significantly different from TPG-treated NS siRNA-transfectants by one-way ANOVA followed by Bonferroni’s multiple comparison test (p < 0.01). (**d**) Western blotting analyses of stress-related protein expression. Total cell lysates prepared 16 h after exposure to 0.2 μg/ml TPG were analyzed using the indicated antibody probes. Cropped blots are shown; all gels were run under the same experimental conditions. Raw data are shown in Supplementary Fig. 6d with dividing lines and with quantitative data.

Lastly, we investigated the effect of *Mtor* knockdown on NMD activity. Knockdown of *Mtor* in C2C12-DMPK160 cells caused significant upregulation of *Hnrnpl_NMD* mRNA compared to non-silencing siRNA-transfectants treated with TPG (Fig. 6a). Western blot analysis demonstrated downregulation of pUPF1, SMG7, and UPF1 compared to TPG-treated, non-silencing siRNA-transfectants. The observed downregulation of pUPF1 suggested NMD suppression under these conditions (Fig. 6b). In addition, knockdown of *Mtor* caused significant upregulation of *Atf4* mRNA compared to TPG-treated, NS siRNA-transfectants (Fig. 6c). However, *Mtor* knockdown induced less ATF4 protein accumulation than NS siRNA-transfection under mild ER stress, despite the upregulation of eIF2α phosphorylation (Fig. 6d). These results suggest that the mTOR pathway affects ATF4 accumulation by an eIF2α phosphorylation-independent mechanism, as previously reported^34,35^.

**Figure 7:**
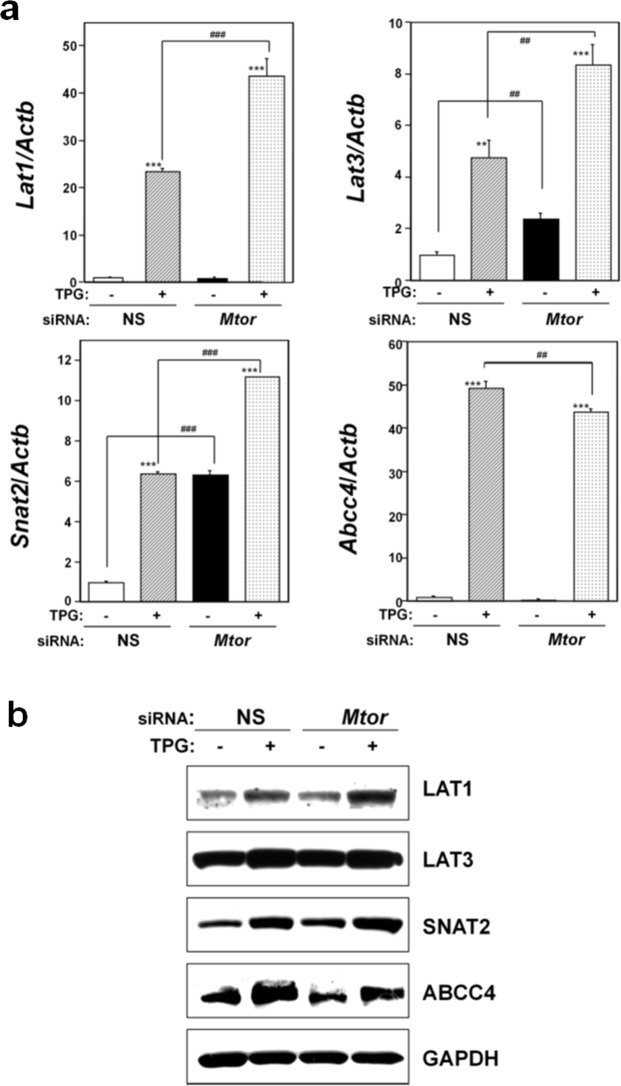
Effect of *mTOR* knockdown on expression of membrane transporters. (**a**) Analyses for membrane transporter mRNA expression by RT-qPCR. Total RNA was extracted from cells that were untreated or treated with 0.2 μg/ml TPG for 16 h. The histogram depicts the indicated mRNA normalized to *Actb* mRNA presented as the fold-increase over non-pretreated NS siRNA-transfectants. Values shown are the mean ± SE of four separate experiments. **^,^***Significantly different from TPG-untreated cells by one-way ANOVA followed by Bonferroni’s multiple comparison test (**p < 0.01, ***p < 0.001). ^##,###^Significantly different from NS siRNA-transfectants by one-way ANOVA followed by Bonferroni’s multiple comparison test (^##^p < 0.01, ^###^p < 0.001). (**b**) Western blots for membrane transporters in C2C12-DMPK160 cells transfected with NS, *mTOR* siRNA. Cropped blots are shown; all gels were run under the same experimental conditions. Raw data are shown in Supplementary Fig. 7b with dividing lines and with quantitative data.

We have previously demonstrated that ATF4 accumulation leads to the upregulation of membrane transporters, including L-type amino acid transporter (LAT) 1, sodium-coupled amino acid transporter 2 (SNAT2), and the ATP-binding cassette transporter cassette C subfamily 4 (ABCC4) expression, but not LAT3^29^. Further, we have shown that NMD suppression leads to the upregulation of LAT1, LAT3, SNAT2, and ABCC4 expression under mild ER stress through ATF4 expression^29^. Therefore, we investigated the role of mTOR pathway in the expression of these genes. As shown in Fig. 7a, *Mtor* knockdown significantly increased mRNA levels of *Lat1*, *Snat2*, and *Lat3*, but not *Abcc4*, in TPG-pretreated cells compared to TPG-treated, NS siRNA-transfectants. Western blot analysis demonstrated upregulation of LAT1 and SNAT2, but not ABCC4 in TPG-treated *Mtor* knockdown cells compared to NS-transfectants (Fig. 7b and Supplemental Fig. 7b). Expression of such membrane transporters might be related to *mTOR* knockdown-induced NMD suppression and lowered ATF4 protein accumulation under mild ER stress.

## Discussion

In this study, we demonstrated that the environmental stresses of oxidative stress and mild ER stress suppress NMD in mouse MeHg-susceptible myogenic cells, rat CNCs, and rat AGCs (Fig. 1). The mechanism for this environmental stress-induced NMD suppression involves eIF2α phosphorylation and stress-induced reduction of the NMD components UPF1, SMG7, and eIF4A3. Phosphorylation of eIF2α, which is a central event in integrated stress responses, has been reported under a variety of stress conditions including hypoxia, ER stress, amino acid deprivation, oxidative stress, and the accumulation of cytokines^15,36,37^. Further, phosphorylation of eIF2α is necessary for the formation of stress granules^38^, which play a role in the accumulation of non-translated mRNAs, translation initiation components, and related proteins during a stress response^39^. It has been reported that NMD suppression is most likely mediated by phospho-eIF2α-mediated suppression of translation in ER-stressed^32^ or hypoxic cells^15^. However, the question of whether eIF2α phosphorylation acts exclusively to suppress NMD under environmental stresses remains unanswered. As shown in Fig. 3e and f, repression of translation caused by eIF2α phosphorylation played a critical role in NMD suppression under the two environmental stresses. However, it was not an exclusive factor because upregulation of NMD target *Hnrnpl_NMD* mRNA and decreased p-UPF1 were also observed in phospho-eIF2α-deficient SA cells under mild ER stress.

**Figure 8:**
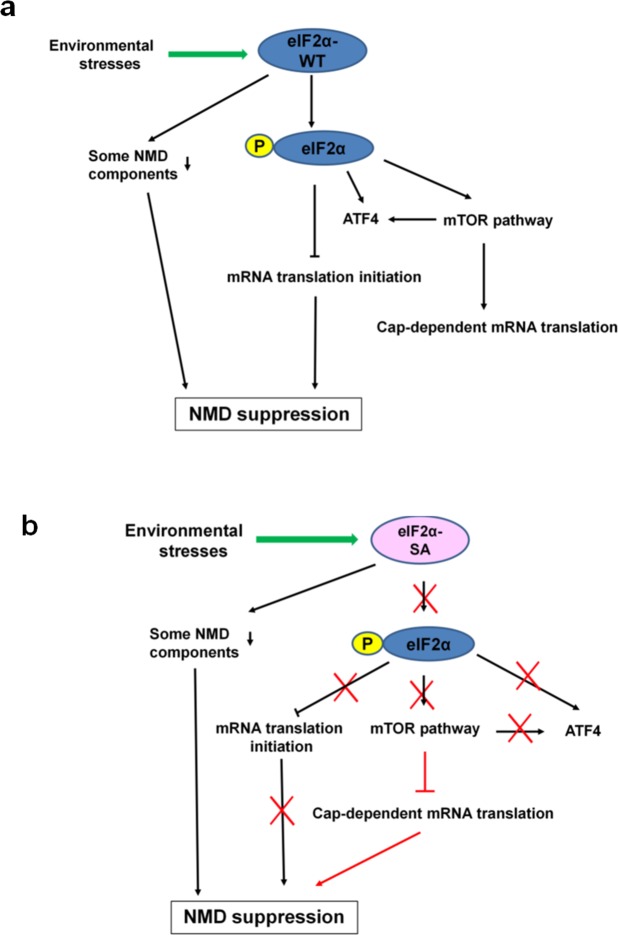
Summary of the interactions among causative factors for mild ER stress-induced NMD suppression. (**a**) WT, stable cell line transfected with wild-type eIF2α plasmid. Phospho-eIF2α-mediated translation suppression plays a critical role for mild ER stress-induced NMD suppression. In addition, reductions of NMD components UPF1, SMG7, and eIF4A3 are involved in the inhibition of NMD. (**b**) SA, stable cell line transfected with mutant eIF2α-SA plasmid. Suppression of NMD in the preconditioned mutant SA cell line is due to mTOR suppression-induced inhibition of cap-dependent translation. In addition, reductions of the NMD components UPF1, SMG7, and eIF4A3 are involved in the inhibition of NMD.

Here, mTOR suppression-induced inhibition of cap-dependent translation contributed to NMD suppression in phospho-eIF2α-deficient SA cells under mild ER stress (Fig. 4a–d). The mTOR pathway is a central controller of cell growth and metabolism in response to environmental cues, such as nutrient limitation, and other types of stresses^40^. The cause-and-effect relationship between the mTOR pathway and NMD activity has been variously reported using rapamycin at different working doses and exposure times in various cells^41–43^. In this study, we demonstrated significant downregulation of mTOR expression in mutant SA-preconditioned cells compared to a control WT cell line (Fig. 4a,b). The rescue experiment involving the activation of mTORC1 using the mTOR activator, MHY1485, in mutant SA-preconditioned cells confirmed the cause-and-effect relationship between the mTOR downregulation and NMD suppression in this cell line (Fig. 4c,d). In addition, we confirmed NMD suppression in mTOR knockdown cells under mild ER stress (Fig. 6a,b). In contrast, mTOR was upregulated in *Atf4* or NMD component knockdown cells under mild ER stress compared to NS siRNA-transfectants (Fig. 5c–f), suggesting that ATF4 accumulation or NMD suppression is not an upstream activator of the mTOR pathway under mild ER stress. We observed reduced ATF4 protein accumulation following *Mtor* knockdown despite the upregulation of eIF2α phosphorylation under mild ER stress, indicating that the mTOR pathway affects ATF4 accumulation by an eIF2α phosphorylation-independent mechanism (Fig. 6c,d). This finding is consistent with recent reports^34,35^ on the relationship between mTORC1 and ATF4. In these studies, mTORC1 affected ATF4 mRNA translation through 4EBP1^34^ and mTORC1-driven eIF4F activity^35^. The environmental stress-induced pathway to NMD suppression we observed presently is summarized in Fig. 8.

The mTOR pathway is related to cellular amino acid metabolism. Here, we demonstrated the upregulation of methionine transporters in *Mtor* knockdown, TPG-pretreated cells compared to NS siRNA-transfectants (Fig. 7a,b). A recent report revealed the contribution of mTORC1 to the cellular amino acid supply through post-transcriptional control of ATF4 in HEK293T cells^34^. Our present and recent results^29^ suggest that *Mtor* knockdown-induced NMD suppression under mild ER stress contributes to the expression of methionine transporters and ABCC4, which reside downstream of ATF4.

Notably, the results of a microarray study on NMD knockdown cells suggested that almost of 10% of unmutated mRNAs are regulated by NMD^14^. Several stress-induced genes are NMD targets as a result of PTC-prone attributes. The attributes include uORFs, alternative mRNA splicing introducing nonsense codons^14,15^, and uncharacterized mechanisms. Suppression of NMD that is induced by mild ER stress stabilizes NMD-targeted genes and augments the cellular stress responses to protect cells against stresses, such as MeHg exposure^17^. The diverse transcripts induced by NMD suppression include mRNAs that play important parts in mediating the unfolded protein response, integrated stress response, and amino acid transport and metabolism, as well as proto-oncogene mRNAs^14^. Our recent study demonstrated that NMD suppression under ER stress preconditioning also upregulates membrane transporter mRNAs related to the influx and efflux of MeHg, leading to a decrease in intracellular mercury content^29^. Further, NMD suppression induced by environmental stresses may affect phenotypes of PTC-related diseases^44,45^. The foregoing suggest that stress-induced NMD suppression has the potential to affect the condition of cells under environmental stress by stabilizing NMD-targeted gene expression.

In conclusion, we demonstrate that environmental stresses suppress NMD in three cell types. Phospho-eIF2α-mediated repression of translation plays a critical role, but is not the sole factor. We also revealed the involvement of mTOR suppression-induced inhibition of cap-dependent translation and downregulation of NMD components UPF1, SMG7, and eIF4A3 in stress-induced NMD suppression.

## Materials and Methods

### Cell culture and drug treatments

C2C12-DMPK160 cells were cultured in Dulbecco’s modified Eagle’s medium (DMEM) (Nissui Pharmaceuticals) supplemented with 10% fetal bovine serum (FBS) (HyClone), 300 μg/ml glutamine (Nissui Pharmaceuticals), and 0.4 mg/ml Geneticin (G418; Thermo Fisher Scientific). A primary culture of rat CNCs was established as described previously^46^ and cultured in Neurobasal medium (Invitrogen). A primary culture of rat AGCs was established on the basis of the previous paper^46^ and cultured in DMEM supplemented with 10% FBS and 300 μg/ml glutamine. Stable cell lines expressing non-phosphorylatable or phosphorylatable human eIF2α^29^ were cultured in DMEM supplemented with 10% FBS and 300 μg/ml glutamine. Exposure to MeHg was carried out in serum-free Cosmedium (Cosmo Bio Co., Ltd) for all cells except CNCs as described previously^19,20^. CNCs were exposed to MeHg in Neurobasal medium. Cell viability for C2C12-DMPK160 cells and AGCs exposed to MeHg is shown in Supplemental Fig. 9. Cell viability data for CNCs has been demonstrated previously^46^. MeHg was dissolved in Ca^2+^- and Mg^2+^-free PBS containing equimolar L-cysteine (WAKO) to enhance the rate of MeHg uptake through the amino acid transport system^47^. TPG (Sigma-Aldrich) was prepared as described previously^17^. For the preconditioning study, 0.2 µg/ml TPG was added to the cells for 16 h before MeHg treatment. After removal of TPG, cells were exposed to MeHg as described previously^19^. MHY1485 (Sigma-Aldrich) stock was dissolved in dimethylsulfoxide. MHY1485 was treated for 3 h for RNA analysis and for 5 h for protein analysis.

### RT-qPCR

Total RNA was extracted, and first-strand cDNA was prepared as described previously^5^. qPCR was performed using a LightCycler DX 400 System (Roche). An SYBR Green Master Mix (Roche) and specific primer sets were used to amplify mRNAs. Specific primer sets for mouse *Snhg1*, *Atf4*, *Lat1*, *Lat3*, *Snat2*, and *Abcc4* have been described previously^17,29^. Primer sets for mouse *Hnrnpl_NMD, Hnrnpl, Tra2b_NMD*, and *TRa2b* were synthesized as reported previously^32^. Primer sets for rat *Gas5* and mouse *Mtor* (Gene Bank accession numbers: NR_002704 and NM_020009, respectively) were as follows: *Gas5* 5′-GCTAAGGACTCATGAGGAAG-3′ (nucleotides 96–115) and 5′-GAAATGAAGGACCTTGTGTG-3′ (nucleotides 284−265); *Mtor* 5′-CCAGGATACACTAAGAGTCC-3′ (nucleotides 5820-5839) and 5′-CGACCAATATCTGTGAGAAG-3′ (nucleotides 6017-5998). Transcript levels were normalized to *Actb* mRNA as described previously^20^.

### Western blot analysis

Western blotting was performed as previously described^44^. Briefly, cells were incubated on ice for 10 min in Cell Lysis buffer (Cell Signaling Technologies) containing complete protease inhibitor cocktail (Roche). Subsequently, the cells were harvested and homogenized using a QIA shredder. Protein content was measured using a DC protein assay kit (Bio-Rad Laboratories). Next, samples were separated in the presence of dithiothreitol (Sigma-Aldrich) by 10% sodium dodecyl sulfate-polyacrylamide gel electrophoresis (SDS-PAGE) for analysis of eIF2α, phospho-eIF2α, ATF4, eIF4A3, LAT1, LAT3, SNAT2, and glyceraldehyde 3-phosphate dehydrogenase (GAPDH), and by 12.5% SDS-PAGE for phospho-4EBP1. For the components of NMD (phospho-UPF1, UPF1, and SMG7), PERK, mTOR, and ABCC4, 5% SDS-PAGE was adopted. The gels were transferred to nitrocellulose membranes (Bio-Rad Laboratories). Membranes were blocked in EzBlock Chemi (ATTO) for 30 min, and then incubated with the following indicated antibody probes: anti-eIF2α, anti-phospho-eIF2α, anti-ATF4, anti-phospho-4EBP1, anti-PERK, anti-mTOR, and anti-GAPDH (Cell Signaling Technologies); anti-eIF4A3, anti-SNAT2, and anti-ABCC4 (Abcam); anti-LAT1 (Sigma-Aldrich); and anti-LAT3 (Nobus Biologicals). Antibodies to the NMD components SMG-1, SMG7, UPF1, and pUPF1 have been described elsewhere^30,31,45,48^. The proteins were detected as described previously^44^. Blots were stripped in the stripping buffer including 62.5 mM Tris-HCl (pH 6.8), 2% SDS, and 100 mM 2-mercaptoethanol. Then, the membrane was reused for re-probing with other antibodies. Densitometric quantification of the immunoblots was performed using NIH Image software.

### siRNA preparation and transfection

Mouse *Smg1* and *Smg7* siRNAs have been described previously^5^. Mouse *Eif2α* (target sequence: 5′-CAAUGUUGUUAUGUUCU-3′) siRNA was designed using the i-Score Designer^49^ and asymmetric siRNA^50^ was synthesized (GeneDesign, Inc.). Other siRNAs, including mouse *Atf4*, FlexiTube siRNA (SI02674427), mouse *Perk*, FlexiTube siRNA (SI01319269), mouse Mtor, FlexiTube siRNA (SI03099796), and All Star Negative Control siRNA were purchased from Qiagen. Transfections of synthetic siRNAs were carried out with Lipofectamine RNAiMAX (Thermo Fisher Scientific) and the cells were analyzed 48 h after transfection. The results were confirmed in more than three independent experiments.

### Statistical analyses

Statistical analyses were conducted using Graph Pad PRISM 5.0 (GraphPad Software). Data were analyzed by one-way ANOVA followed by Bonferroni’s multiple comparison test, or one-way Welch’s *t*-test for dual comparison. The results are expressed as mean ± SEM. A difference was considered statistically significant when p < 0.05.

## Supplementary information

Supplementary Figures
