# Supplementary material for: Environmental stresses suppress nonsense-mediated mRNA decay (NMD) and affect cells by stabilizing NMD-targeted gene expression
Source: Sci Rep. 2019 Feb 4;9:1279. doi: 10.1038/s41598-018-38015-2 (PMC6362056; doi:10.1038/s41598-018-38015-2)
Supplement: Supplementary file 1 — Supplementary Figures [file 41598_2018_38015_MOESM1_ESM.pdf]

Environmental stresses suppress nonsense-mediated mRNA decay (NMD) and affect cells by stabilizing NMD-targeted gene expression

Fusako Usuki[1,*], Akio Yamashita[2], Masatake Fujimura[3]

[1]Department of Clinical Medicine, [3]Basic Medical Sciences, National Institute for Minamata Disease, 4058-18 Hama, Minamata 867-0008, Japan

[2]Department of Molecular Biology, Yokohama City University School of Medicine, 3-9 Fuku-ura, Kanazawa, Yokohama 236-0004, Japan

[*]To whom correspondence should be addressed. Tel: +81 966 63 3111; Fax: +81 966 61 1145; E-mail: usuki@nimd.go.jp

Supplenmantary Fig.1c

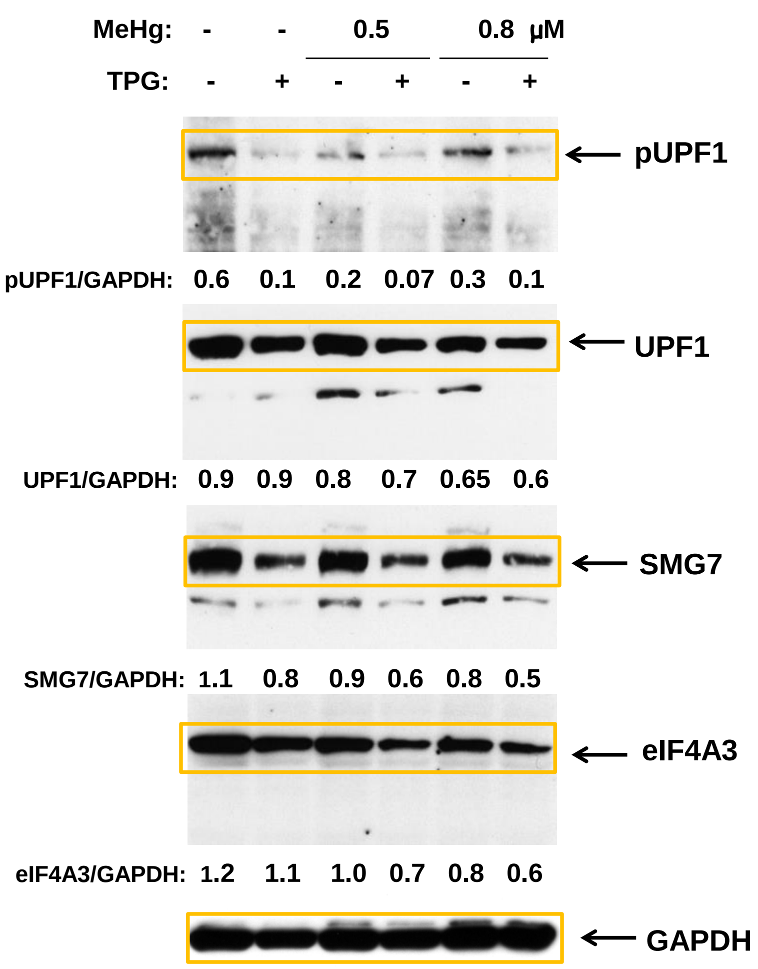

Supplenmantary Fig.1e

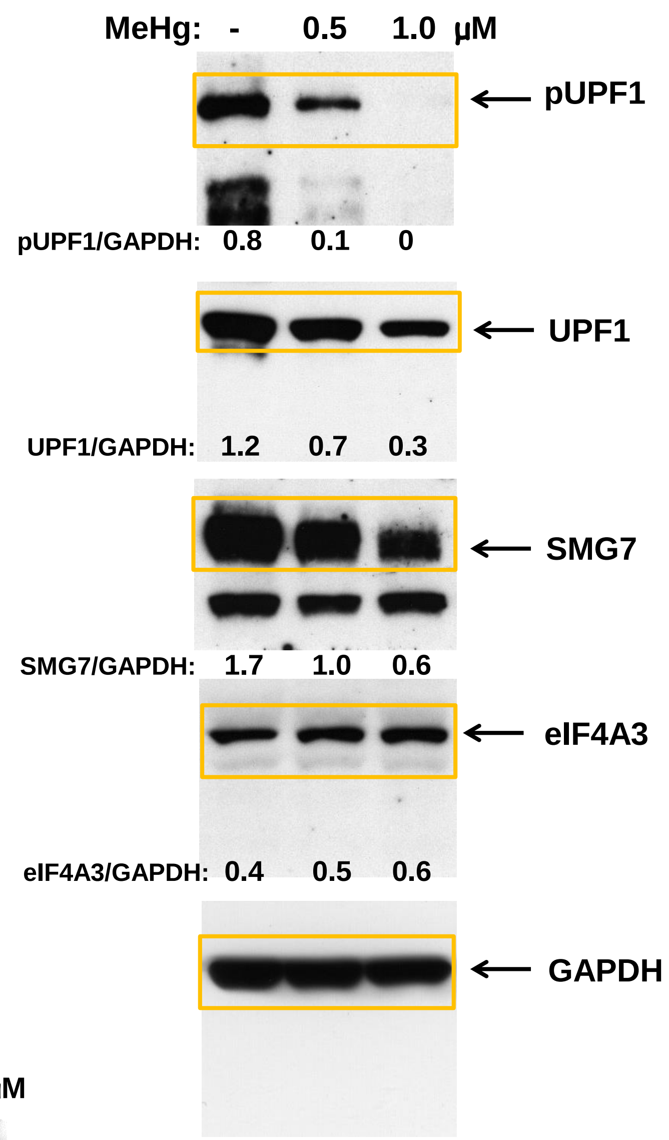

Supplenmantary Fig.1g

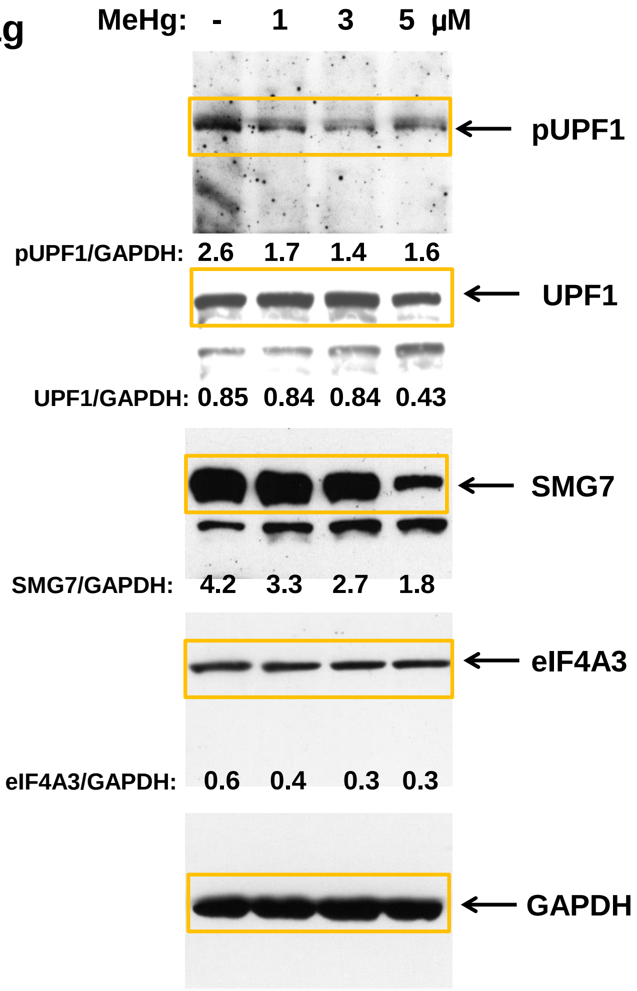

Supplenmantary Fig. 3b

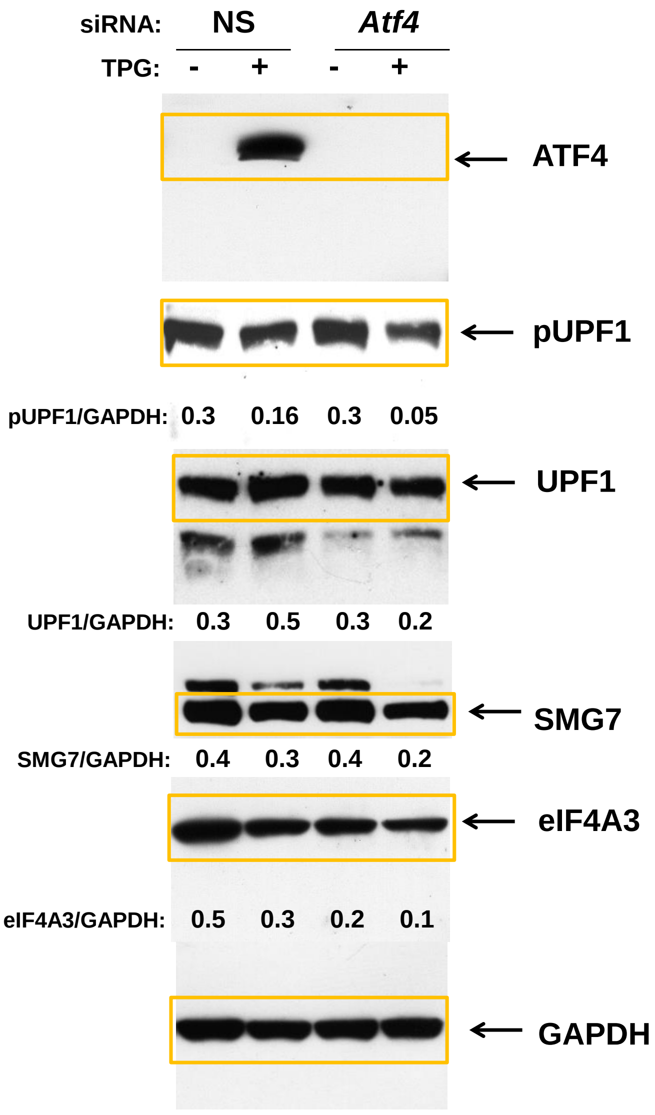

Supplenmantary Fig. 3d

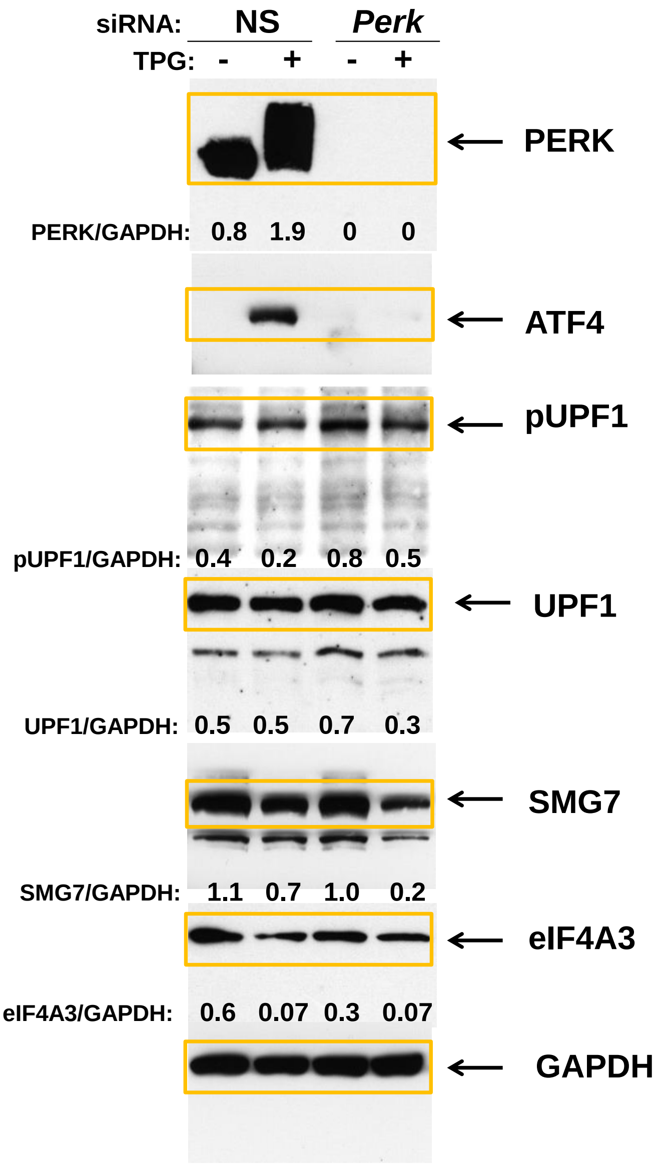

Supplenmantary Fig. 3f

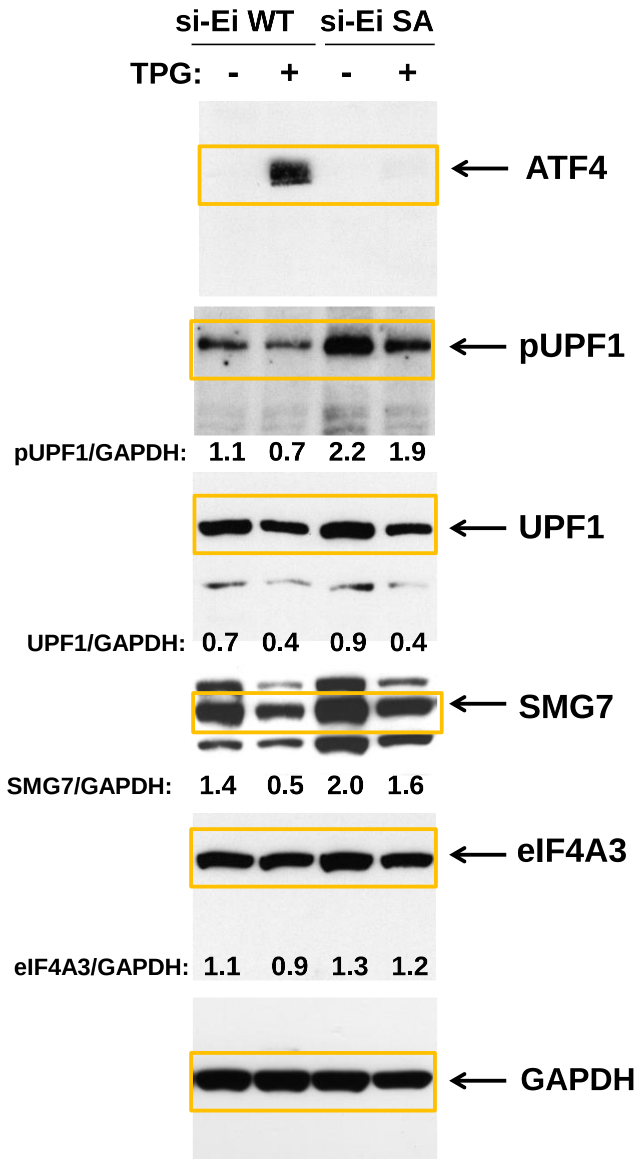

Supplenmantary Fig. 4b

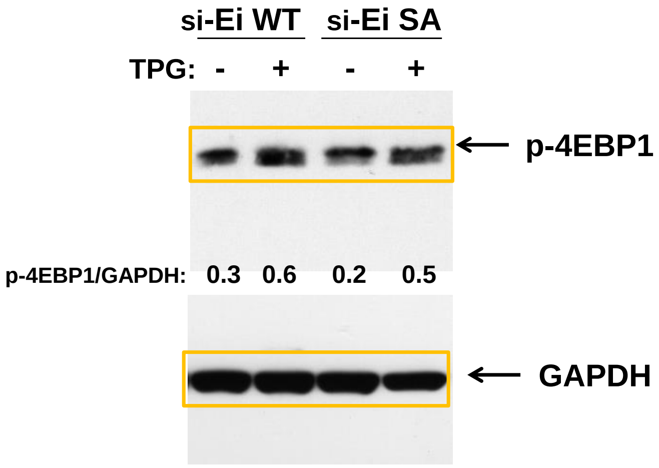

Supplenmantary Fig. 4c

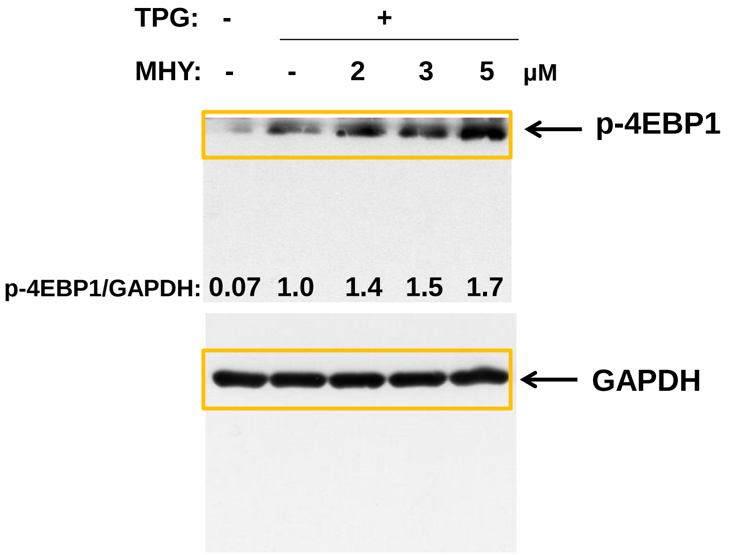

Supplenmantary Fig. 5b

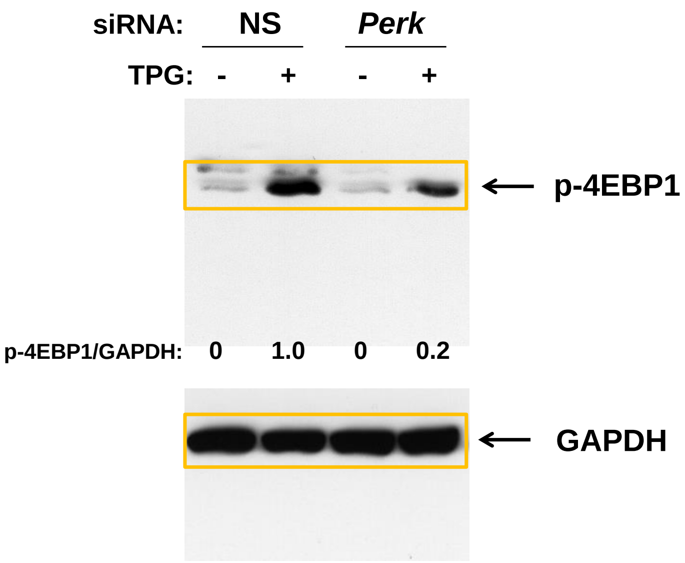

Supplenmantary Fig. 5d

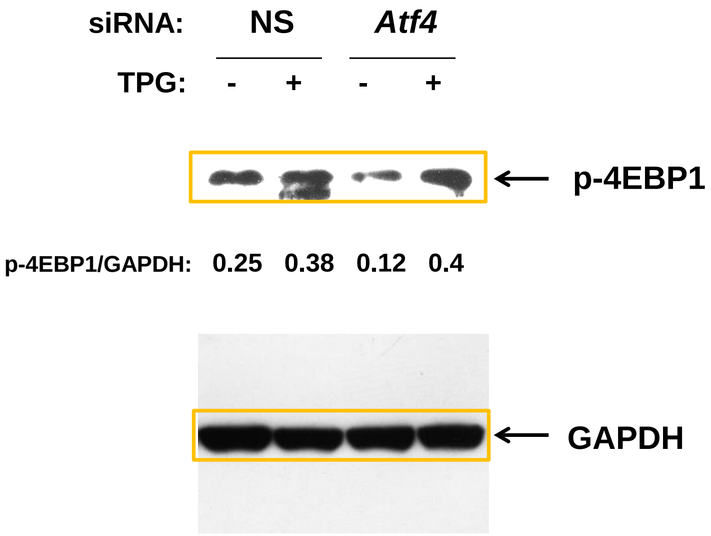

Supplenmantary Fig. 5f

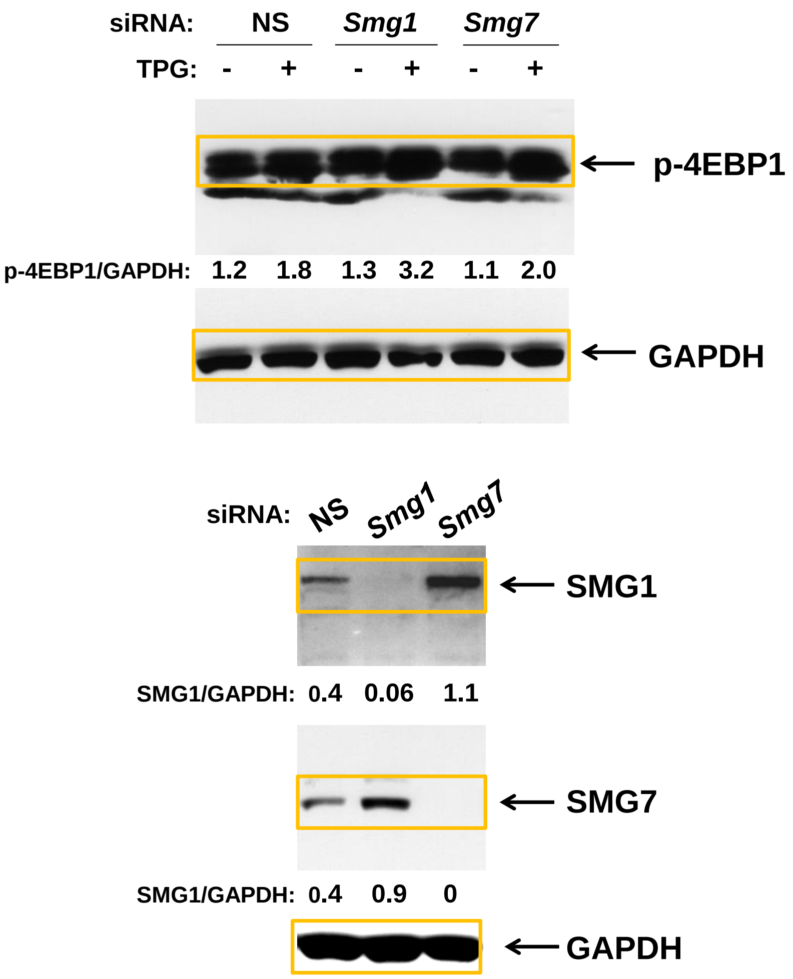

Supplenmantary Fig. 6b

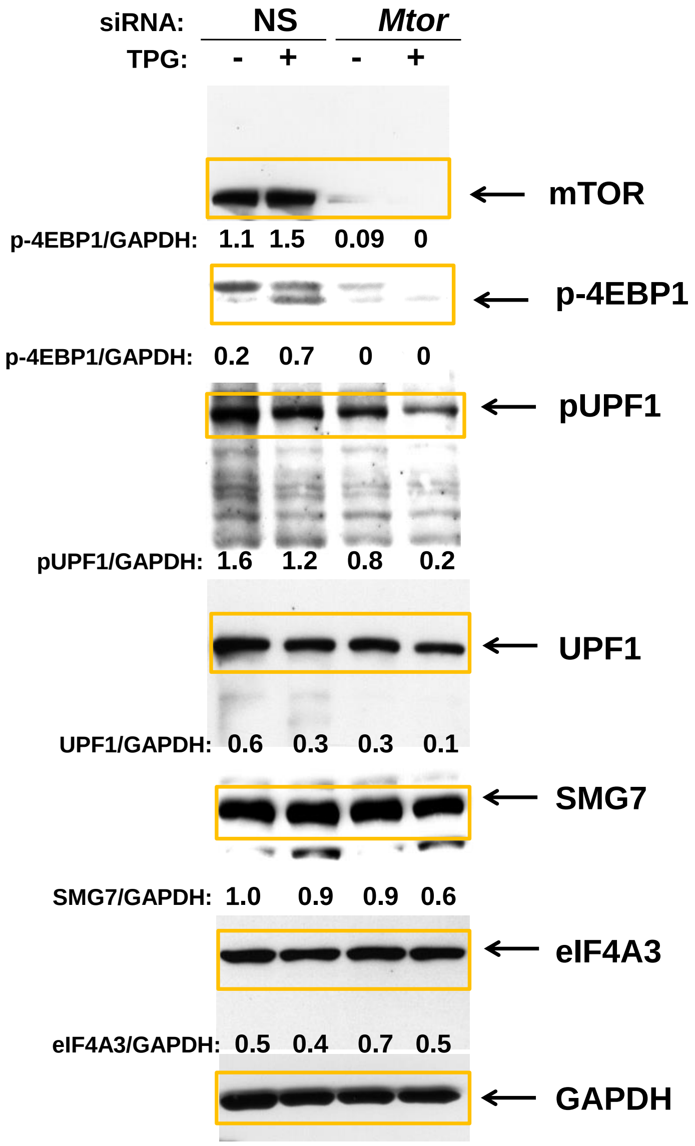

Supplenmantary Fig. 6d

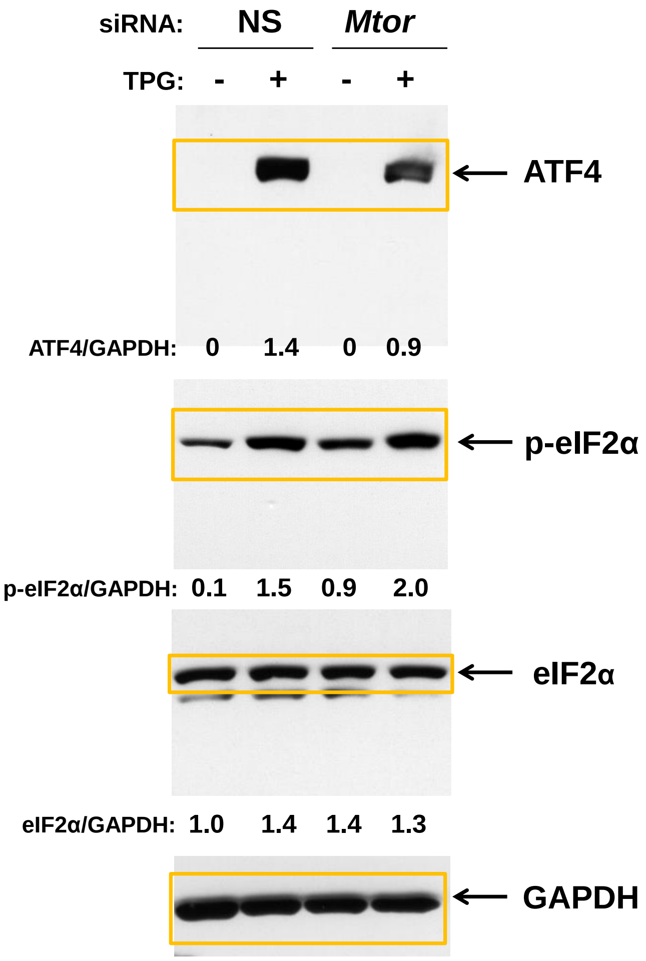

Supplenmantary Fig. 7b

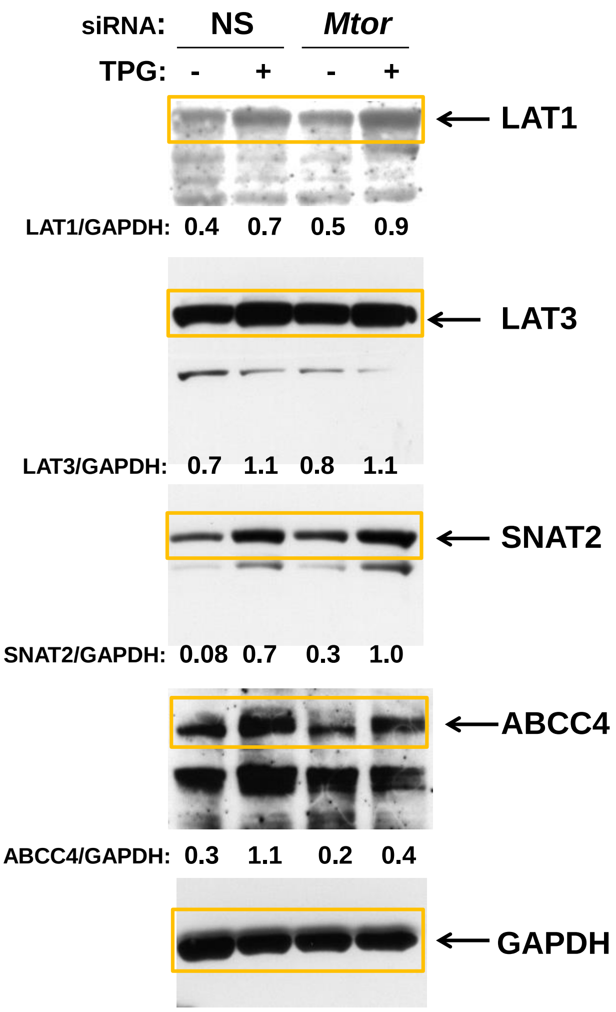

Supplenmantary Fig.9

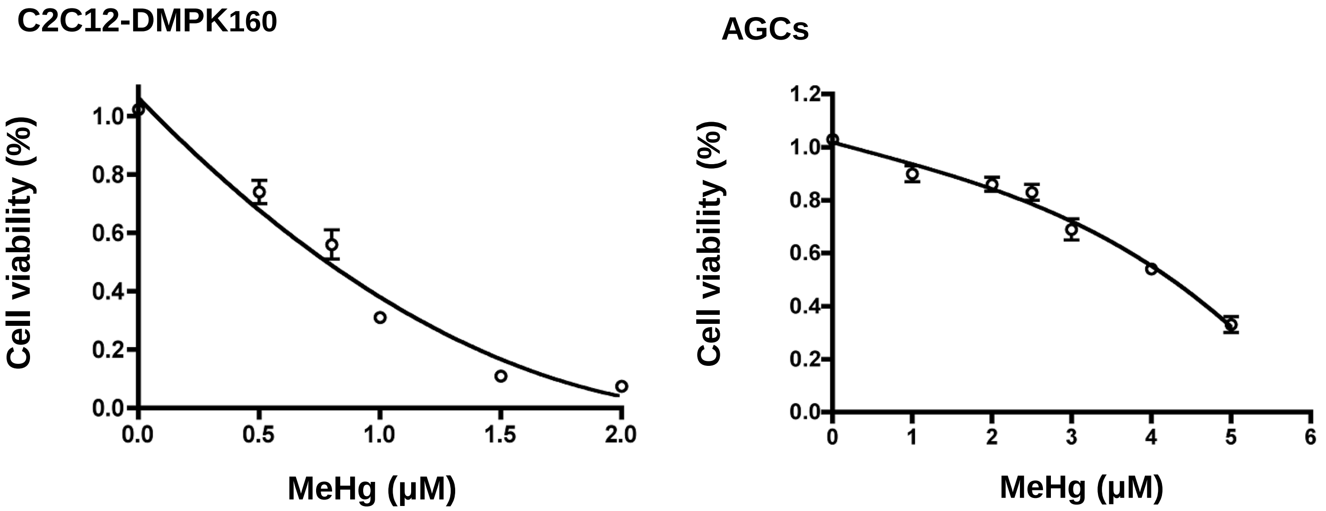

Effects of MeHg on the viability of C2C12-DMPK160 cells or AGCs. Viability was determined with a Cell Counting Kit (DOJINDO) using the dye WST-1 24 h after exposure to MeHg. The viability of untreated cells was regarded as 100%. Values represent the mean ± SD (n = 6).
